# Stability and Harmony of Gait in Patients with Subacute Stroke

**DOI:** 10.1007/s40846-016-0178-0

**Published:** 2016-10-07

**Authors:** Marco Iosa, Fabiano Bini, Franco Marinozzi, Augusto Fusco, Giovanni Morone, Giacomo Koch, Alex Martino Cinnera, Sonia Bonnì, Stefano Paolucci

**Affiliations:** 1 IRCCS Fondazione Santa Lucia, Via Ardeatina 306, 00179 Rome, Italy; 2 Aerospace and Mechanical Engineering Department, Sapienza University of Rome, 00184 Rome, Italy; 3 Stroke Unit, Policlinico Tor Vergata, 00133 Rome, Italy

**Keywords:** Ambulation, Biomechanics, Balance, Rehabilitation, Accelerometry, Golden ratio, Walking

## Abstract

Stroke affects many gait features, such as gait stability, symmetry, and harmony. However, it is still unclear which of these features are directly altered by primary damage, and which are affected by the reduced walking speed. The aim of this study was to analyze the above gait features in patients with subacute stroke with respect to the values observed in age- and speed-matched healthy subjects. A wearable triaxial accelerometer and an optoelectronic device were used for assessing the upright gait stability, symmetry of trunk movements, and harmonic structure of gait phases by means of the root-mean-square (RMS) acceleration of the trunk, harmonic ratio (HR), and gait ratios (GRs), respectively. For healthy subjects, results showed that RMS acceleration increased with speed, HR peaked at a comfortable speed, and GRs tended towards the theoretical value of the golden ratio for speeds >1 m/s. At matched speed conditions, patients showed higher instabilities in the latero-lateral axis (*p* = 0.001) and reduced symmetry of trunk movements (*p* = 0.002). Different from healthy subjects, antero-posterior and latero-lateral acceleration harmonics were coupled in patients (R = 0.507, *p* = 0.023). Conversely, GRs were not more altered in patients than in slow-walking healthy subjects. In conclusion, patients with stroke showed some characteristics similar to those of the elderly when the latter subjects walk slowly, and some altered characteristics, such as increased latero-lateral instabilities coupled with movements performed along the antero-posterior axis.

## Introduction

The walking patterns of patients with stroke were extensively analyzed quantitatively in the 1990s using instrumented systems for gait analysis based on stereophotogrammetry, force platforms, electromyography, and metabolimeters [1–3]. Classical gait analysis provides a huge quantity of data, usually focused on hip, knee, and ankle angular kinematics and kinetics, but it is expensive and time-consuming in clinical settings. There is thus a need for a few meaningful indices for assessing deterioration or amelioration of patients’ gait [4].

More recently, many researchers have started to use less-expensive wearable and optoelectronic devices [5], devoting more attention to the entire locomotor system, including the upper body (not only lower limbs) [6], and highlighting the need of measuring few clinically meaningful benchmarks of human gait, such as stability, symmetry, and harmony [7, 8].

As described in a recent review [6], “stable gait” may refer to the repeatability of walking [9], gait resilience to perturbations [10], or the ability to maintain upright balance during walking [11]. This last aspect (denoted as upright gait stability [6]) is one of the most important ones for patients with stroke because of their high risk of falls [12].

The upright gait stability of patients with stroke has been investigated by computing the root-mean-square (RMS) accelerations of the upper body [13, 14]. However, an increase in upper body accelerations could be attributed to an unsteady speed (for example, due to pathological instabilities), as well as to an increase in walking speed (WS), which is commonly related to a clinical amelioration [13]. For this reason, trunk acceleration usually needs to be normalized by velocity in order to allow a comparison between populations walking at different speeds.

Another important aspect of gait that is increasingly receiving attention is harmony. Harmonic gait may refer to different features of human walking. The most commonly used parameter is the so-called harmonic ratio (HR), which is related to the bilateral rhythmicity of movement, based on the measure of trunk acceleration during a stride that is expected to be formed by two alternating symmetric steps [15–17]. More specifically, HR is the ratio between the sum of the amplitudes of even harmonics and the sum of the amplitudes of odd harmonics calculated via the discrete Fourier transform along the antero-posterior (AP) and cranio-caudal (CC) directions; the opposite ratio is calculated along the latero-lateral axis (LL). A recent study found that, among many variables, the HR of trunk acceleration was the only gait variable with a good discriminative ability of predicting the incidence of falls among older people [18]. Its use in pathological gait analysis, especially in patients with neurological injuries, is hence a logical progression. A reduced HR has been found in subjects with stroke with respect to age-matched healthy subjects [13]. However, because Menz et al. showed that in healthy subjects HR peaks at a comfortable WS [11], again, normalization by velocity is needed.

Another interesting feature of gait related to harmony has recently been discovered. It is related to the rhythmic structure of gait phases. It has been noted that physiological gait has a relationship with the golden ratio [19]. The golden ratio (ϕ) is an irrational number with specific harmonic iterative properties, and has been found in other biological fields [20–22]. The repetitive phases of each gait cycle were found to be in repetitive proportions within each other in physiological human walking, forming an intrinsic harmonic structure of gait [19]. In fact, the ratios between the entire gait cycle and the stance phase, between stance and swing phase, and even between the swing phase and the total double support phase were all found to be neither statistically different from each other nor ϕ. Although many previous studies reported altered percentages of stance and swing in pathological gait, such as for patients with stroke [23], Parkinson’s disease [24], and muscular dystrophy [25], the gait ratio (GR), which physiologically tends to the golden ratio, has never been directly measured in subjects with stroke.

The main aim of this study was to investigate the upright stability, symmetry and harmony of gait in patients with stroke in the subacute phase, comparing their data with those of a group of healthy subjects matched for age and WS (and hence without the need for mathematical normalization).

## Materials and Methods

### Participants

Thirteen patients with stroke and ten age-matched (*t* test: *t* = 0.037, *df* = 21, *p* = 0.971) healthy subjects were enrolled in this study (Table 1). Sample size was determined according to previous data on antero-posterior accelerations of patients with stroke compared to age-matched healthy subjects [13]: setting the alpha level at 5 % and the power of analysis (1-beta) at 95 %, the minimum number of subjects needed for finding statistically significant differences was at least 10 for each group. All the enrolled patients were in the subacute phase of stroke (time from acute event ≤6 months), were able to walk autonomously, and their mean Barthel Index was 83 ± 13 (the Barthel Index is a commonly used clinical scale for assessing the independency of daily living activities; scores 0 and 100 represent total dependency and total independency, respectively [26]).

**Table 1:** Demographics and clinical features of patients (P_i_), averaged in patients’group (PG) for comparisons with healthy subjects’ group (HG), with relevant means ± standard deviations reported in last two rows of table

Subjects	Age (years)	Type of stroke	Hemiparesis	Time since stroke (months)	Barthel Index
P1	51	Ischemic	Left	1	95
P2	70	Ischemic	Right	1	60
P3	74	Ischemic	Left	2	80
P4	43	Hemorrhagic	Left	2	85
P5	63	Ischemic	Right	3	95
P6	64	Hemorrhagic	Right	3	100
P7	50	Ischemic	Right	2	100
P8	75	Hemorrhagic	Right	1	95
P9	75	Ischemic	Left	5	65
P10	72	Ischemic	Left	1	75
P11	66	Ischemic	Left	6	80
P12	65	Ischemic	Right	4	70
P13	62	Ischemic	Left	6	80
PG	63.85 ± 10.24	77 % ischemic	54 % left	2.85 ± 1.86	83 ± 13
HG	63.70 ± 8.00	–	–	–	–

### Experimental Setup

Participants were asked to stand on a line marked on the floor and then to walk straight forward until arriving at another line on the floor (7 m away) and then to walk back. They then had to repeat this two more times. All subjects were initially asked to walk at a comfortable speed. Then, only healthy subjects were asked to repeat the test walking “slow” and “fast”. The fast speed was not expected to be matched with that of patients, but this condition was included for a deeper analysis of the relationship between gait features and speed in physiological conditions.

For each condition, at least eight steps in the middle of the walking pathway were recorded and analyzed for each subject. Spatio-temporal parameters were computed using an optoelectronic system (Optogait^®^, Microgate, Italy; sampling frequency = 100 Hz) placed on the ground in our laboratory and comprising electronic bars containing an infrared light emitter/receiver (each 1.04 cm). The light emitters and receivers formed on the ground a matrix of 384 × 96 = 36,864 invisible cells (each 1.08 cm^2^), able to detect the spatio-temporal parameters of walking. Furthermore, during the test, participants wore an elastic belt that contained a wearable inertial sensor device (FreeSense^®^, Sensorize s.r.l., Italy; sampling frequency = 100 Hz) located on their back corresponding to L2–L3 spinous processes, close to their body center of mass. This device is light-weight (93 g) and contains a tri-axial accelerometer for measuring accelerations along the three body axes (AP, LL, and CC axes). All subjects wore their commonly used shoes during the tests.

### Measurements

The acceleration data recorded during the tests were analyzed after subtracting their mean values and low-pass filtering them at 20 Hz with a fourth-order Butterworth filter [17]. Mean subtraction is a simple approximation often used for roughly compensating for gravitational effects [11, 13, 14].

For assessing upright gait stability, the RMS value of each of the three components of acceleration (one along each body axis) was computed in accordance with the most relevant literature [6, 11, 13, 14]. RMS acceleration, a measure of acceleration dispersion, was computed as follows:

$$RMS = \sqrt {\frac{{\sum\limits_{i = 1}^{N} {a_{i} } }}{N}}$$

where a_i_ is the acceleration measured at the i-th sampled value. It coincides with the standard deviation of accelerometric data because of signal mean subtraction [11, 13, 14].

We then computed HR, which is based on the premise that the unit of measurement for continuous walking is a stride formed by two symmetric steps. Hence, despite its name, HR is a measure of gait symmetry, based on the harmonics of acceleration signals [15]. HR is computed as the ratio between the sum of the amplitudes of even/odd harmonics (calculated via the discrete Fourier transform) of AP and CC components of acceleration or odd/even harmonics for the LL component. The following formulas were used [16, 17]:

$${\text{HR}} = \frac{{\sum\limits_{{{\text{i}} = 1}}^{10} {{\text{A}}_{{{\text{i}}*2}} } }}{{\sum\limits_{{{\text{i}} = 1}}^{10} {{\text{A}}_{{{\text{i}}*2 - 1}} } }}\, {\text{along AP and CC axes}}$$

$${\text{HR}} = \frac{{\sum\limits_{{{\text{i}} = 1}}^{10} {{\text{A}}_{{{\text{i}}*2 - 1}} } }}{{\sum\limits_{{{\text{i}} = 1}}^{10} {{\text{A}}_{{{\text{i}}*2}} } }}\,{\text{along LL axis}}$$

where A_i_ is the amplitude of the first 20 harmonics (subscript “i” is an index used for defining the 10 even and 10 odd harmonics) [11]. To investigate the role of each harmonic, its relative power was computed as the ratio between its amplitude and the sum of the amplitudes of the first 20 harmonics [17].

The information related to the spatio-temporal gait parameters was extracted from the optogait, in particular WS, stride duration, stance phase, swing phase, and double support phase, expressed in percentages of the gait cycle. According to Dzeladini et al. [27], we defined GR_0_ = stride duration/stance duration, GR_1_ = stance duration/swing duration, GR_2_ = swing duration/total duration of double support phases. During physiological gait, these three values should theoretically not be different from each other or ϕ [19].

### Statistical Analysis

The mean and standard deviation of all the described parameters were computed. Analysis of variance (ANOVA) was first applied for assessing in healthy subjects the differences among the three walking conditions (comfortable walking, slow walking, fast walking). Then, patients’ data were compared with those of healthy subjects at matched speed by means of one-way ANOVA (F, relevant degrees of freedom, and *p* values are reported). For the three GRs, we also performed a comparison between values computed for the paretic versus non-paretic limbs using ANOVA.

Quadratic and exponential fits were applied to RMS acceleration and GRs values plotted vs. WS, respectively. Fits were performed using the minimum least squares method. The coefficient of determination of the fit (R^2^) is reported. Pearson’s coefficient (R) was computed for assessing the statistical significance of correlations.

## Results

Table 2 shows the mean results for patients and healthy subjects in the three conditions. The data of healthy subjects were first analyzed to compare the parameters at the three walking conditions, finding significant differences in terms of speed, RMS trunk acceleration, and GRs, but not in terms of HR.

**Table 2:** Means and standard deviations of walking speed (WS, m/s), RMS acceleration (m/s^2^), harmonic ratio (HR, nondimensional number), gait ratio (GR, nondimensional number), and relevant results of ANOVA in terms of F-(degrees of freedom) and *p* values (in bold if statistically significant) among three walking conditions for healthy adults (slow, comfortable, and fast walking) and between patient groups (PG) and slow- or comfortable-walking healthy subjects

Gait feature		Mobility	Upright gait stability			Harmonic of trunk			Harmony of gait cycle phases		
Gait parameter		WS	RMS–CC	RMS–LL	RMS–AP	HR–CC	HR–LL	HR–AP	GR0	GR1	GR2
Healthy adults	Slow	0.64 ± 0.14	0.91 ± 0.34	0.67 ± 0.18	0.86 ± 0.20	2.11 ± 0.73	1.62 ± 0.47	2.32 ± 0.88	1.46 ± 0.07	2.21 ± 0.31	0.87 ± 0.23
	Comfortable	1.01 ± 0.17	1.69 ± 0.56	1.05 ± 0.36	1.30 ± 0.29	2.46 ± 0.50	1.63 ± 0.24	2.58 ± 0.78	1.56 ± 0.05	1.81 ± 0.17	1.25 ± 0.23
	Fast	1.37 ± 0.17	2.70 ± 0.60	1.90 ± 0.61	1.84 ± 0.37	2.03 ± 0.81	1.67 ± 0.49	2.11 ± 0.70	1.62 ± 0.05	1.59 ± 0.13	1.76 ± 0.37
ANOVA	F(2,27)	44.243	30.651	22.410	27.258	1.078	0.032	0.884	20.723	21.816	24.646
	*p*	**<0.001**	**<0.001**	**<0.001**	**<0.001**	0.355	0.969	0.425	**<0.001**	**<0.001**	**<0.001**
Patients	Comfortable	0.67 ± 0.21	1.19 ± 0.33	1.06 ± 0.26	0.97 ± 0.21	1.31 ± 0.34	1.39 ± 0.24	1.33 ± 0.41	1.51 ± 0.04	1.98 ± 0.17	1.07 ± 0.19
PG versus slow	F(1,21)	0.435	3.788	16.325	1.706	12.298	2.359	12.907	5.648	5.612	4.951
	*p*	0.516	0.065	**0.001**	0.206	**0.002**	0.139	**0.002**	**0.027**	**0.028**	**0.037**
PG versus comfortable	F(1,21)	16.202	7.187	0.001	9.739	42.696	5.709	24.493	5.811	5.632	4.454
	*p*	**0.001**	**0.014**	0.970	**0.005**	**<0.001**	**0.026**	**<0.001**	**0.025**	**0.027**	**0.045**

**Fig. 1:**
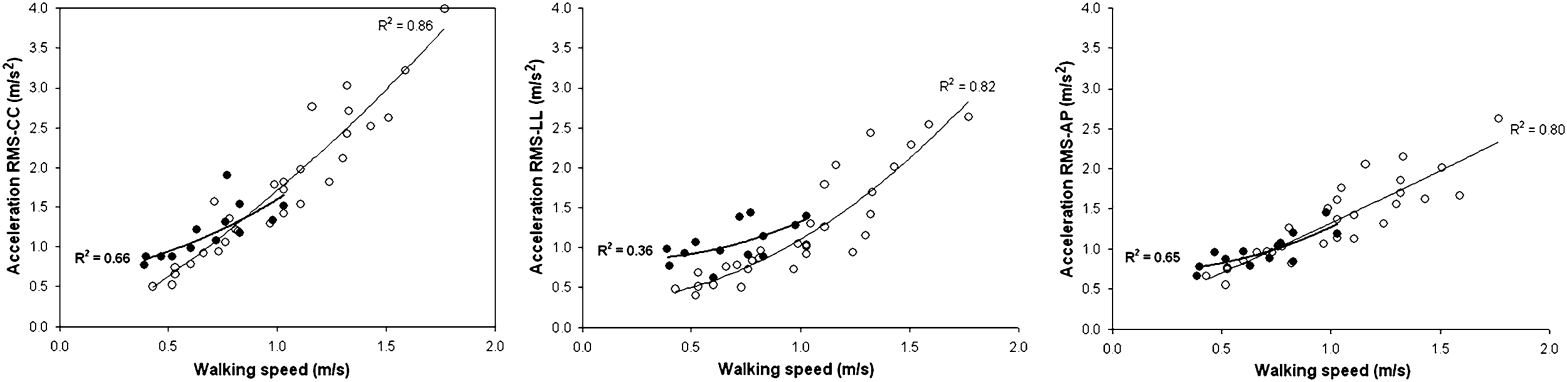
Mean values of RMS acceleration for patients (*filled circles*) and healthy subjects (*empty circles*) and relevant quadratic fits (*bold and thin lines*, respectively) with coefficients of determination along cranio-caudal (CC, *on left*), latero-lateral (LL, *in middle*), and antero-posterior (AP, *on right*) body axes

The speed of patients was significantly lower than the comfortable speed of healthy subjects, but not significantly different from that of healthy subjects during slow walking. In the speed-matched condition, patients showed higher trunk accelerations along the LL axis with respect to those of the slow-walking healthy adults. Interestingly, patients’ RMS acceleration along the LL axis was not different from that of healthy subjects walking at a comfortable speed. Figure 1 depicts the exponential trends of trunk accelerations with respect to WS, with similar trends for RMS accelerations along CC and AP axes between patients and slow-walking healthy subjects, and higher RMS accelerations along the LL axis in patients.

**Fig. 2:**
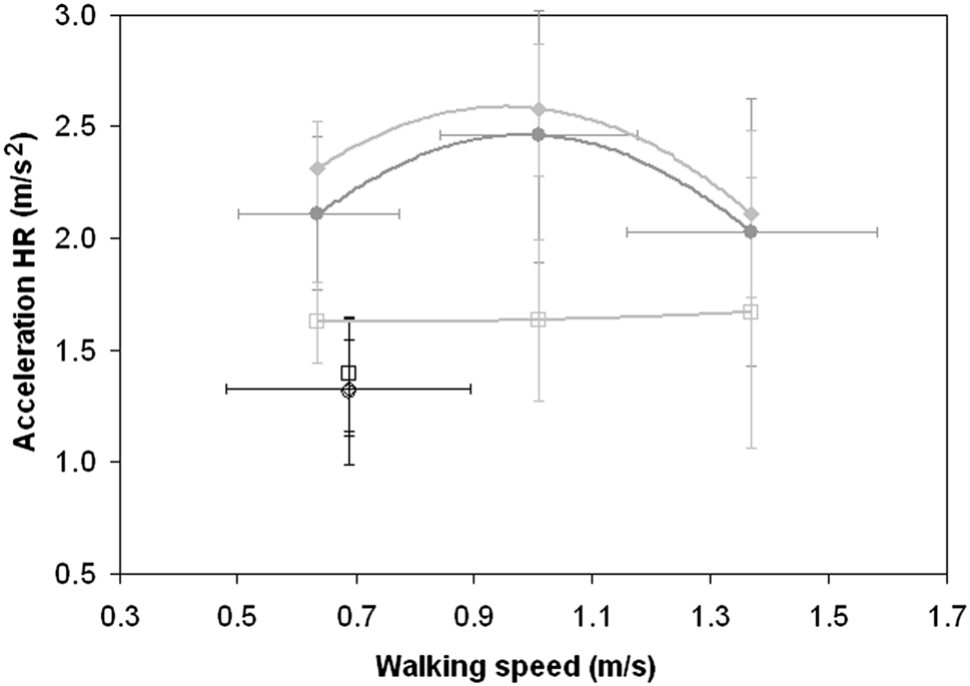
Means and standard deviations (*vertical bars*) of harmonic ratio (HR) along cranio-caudal (CC, *circles*), latero-lateral (LL, *squares*), and antero-posterior (AP, *diamonds*) body axis for healthy adults (*grey*) and patients (*black markers*) reported with respect to the walking condition (slow, comfortable or fast). Walking speed standard deviations (*horizontal bars*) are reported only for CC data of both groups, being the same for the other two axes

Differently from RMS acceleration, the goodness of quadratic fits of HRs with respect to WS for healthy subjects was poor, but statistically significant along CC and AP axes (HR–CC: R^2^ = 0.318, *p* = 0.006; HR–AP: R^2^ = 0.306, *p* = 0.007) but not along the LL axis (R^2^ = 0.127, *p* = 0.860). Due to the poor goodness of fit, Fig. 2 shows only mean values, which clearly have a peak at a comfortable speed for HR–CC and HR–AP. A significant general reduction of HR was found in patients with respect to both slow- and comfortable-speed-walking healthy subjects (see Table 2). Patients’ HR–LL was lower than that of healthy adults when they walked at a comfortable speed, but not for slow walking. Different from healthy subjects (F(2,54) = 64.849, *p* < 0.001), patients’ HR values were not significantly different along the three axes (F(2,24) = 0.337, *p* = 0.717).

**Fig. 3:**
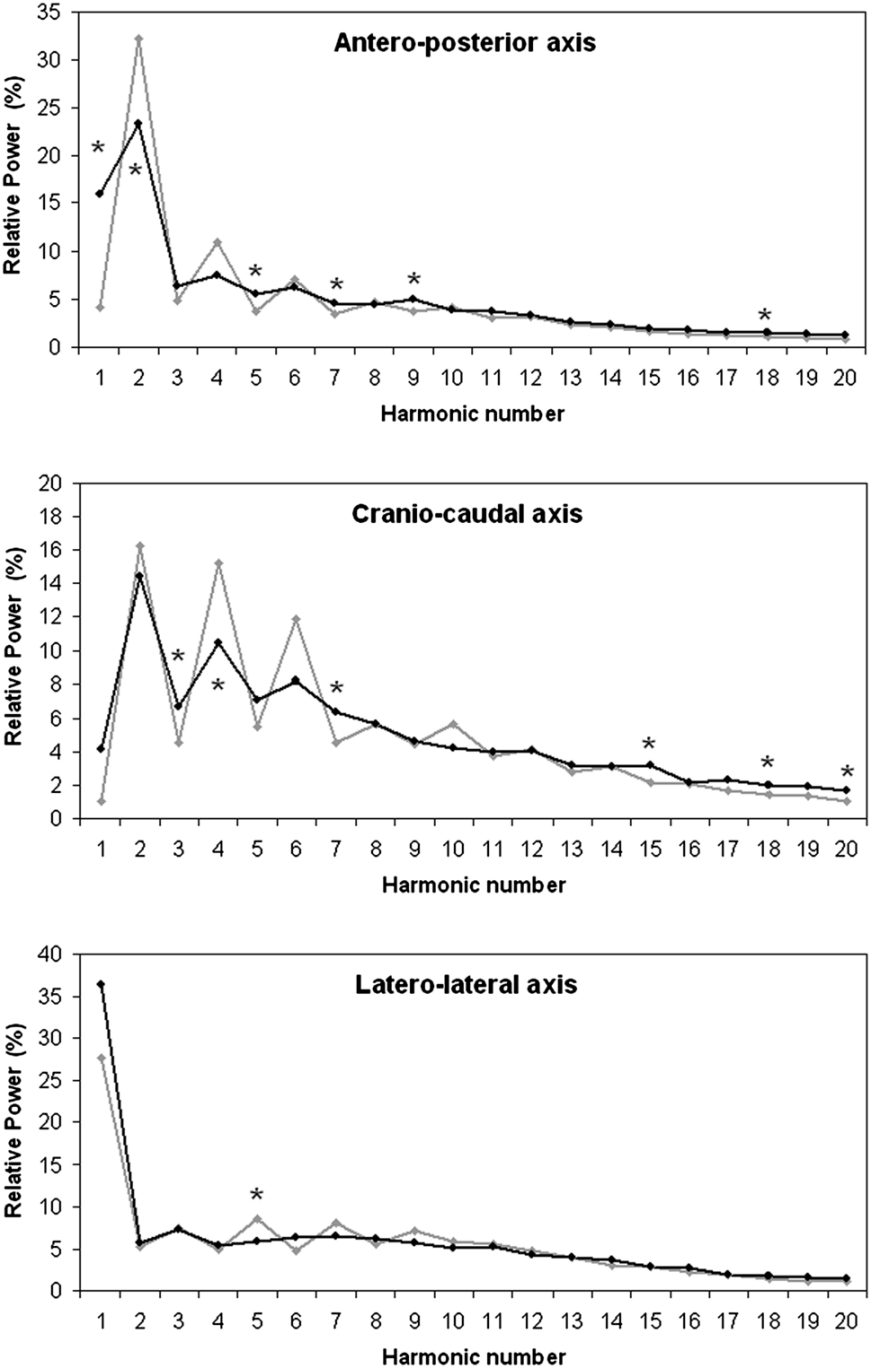
Relative power of first 20 harmonics evaluated along three body axes for patients (*black*) and healthy subjects (*grey*) walking at similar speeds. Stars indicate statistically significant differences

To further analyze these results, the relative power of each harmonic was analyzed, comparing data of patients with those of healthy subjects walking at slow speed. The typical alternation of relative power of odd and even harmonics is shown in Fig. 3. In patients, the relative power of some high-order AP and CC harmonics was increased, whereas among low-order harmonics, the relative power of odd harmonics was increased and that of even harmonics was decreased. Along the LL axis, the only statistically significant difference was found for the 5^th^ harmonic, the power of which was reduced in patients. Furthermore, the physiological alternation of odd and even harmonics already disappeared after the 4th harmonic in patients. For healthy subjects, the relative powers of AP and CC harmonics were significantly correlated with each other (R = 0.822, *p* < 0.001), without any significant correlations between those of AP and LL (R = 0.217, *p* = 0.357) or between those of CC and LL (R = 0.191, *p* = 0.420). For patients, CC and AP harmonic powers were correlated (R = 0.857, *p* < 0.001), and those of LL and AP harmonics were significantly associated (R = 0.507, *p* = 0.023).

**Fig. 4:**
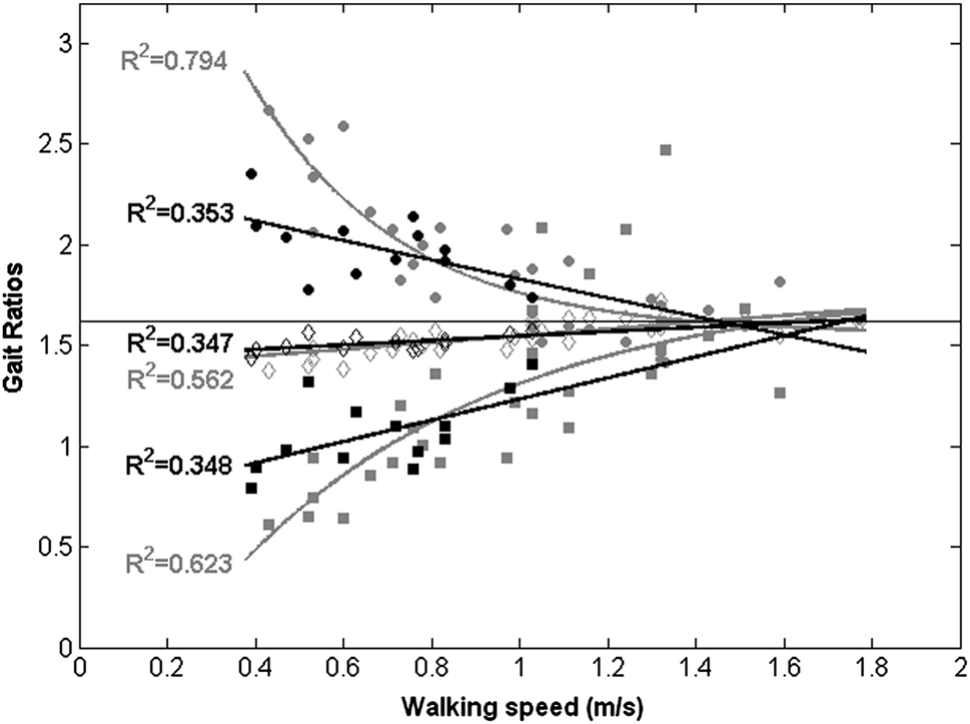
Gait ratios (GR0: *empty diamonds*, GR1: *filled circles*, GR2: *filled squares*) for patients (*black*) and healthy adults (*grey*) with relevant exponential fits. Horizontal line represents theoretical golden gait ratio

The three GRs in healthy subjects were significantly different from each other at slow speed (F(2,18) = 60.543, *p* < 0.001) and comfortable speed (F(2,18) = 20.288, *p* < 0.001), but not at fast speed (F2,18) = 1.313, *p* = 0.294). The three GRs recorded for patients were also significantly different from each other (within-subject factor: F(2,48) = 122.387, *p* < 0.001), but not between paretic and non-paretic limbs (F(1,24) = 0.01, *p* = 0.921). The mean GRs observed for patients were significantly different both from those of slow- and comfortable-speed-walking healthy adults, with values located between them. Figure 4 shows the trend of GRs with respect to speed. Of note, the three GRs for healthy subjects converged towards not significantly different values (and hence towards the theoretical gait golden ratio) for WS > 1 m/s (F(2,30) = 0.091, *p* = 0.914), but not for WS < 1 m/s (F(2,26) = 72.510, *p* < 0.001). The coefficients of determination were lower for patients because of a higher inter-subject variability.

**Fig. 5:**
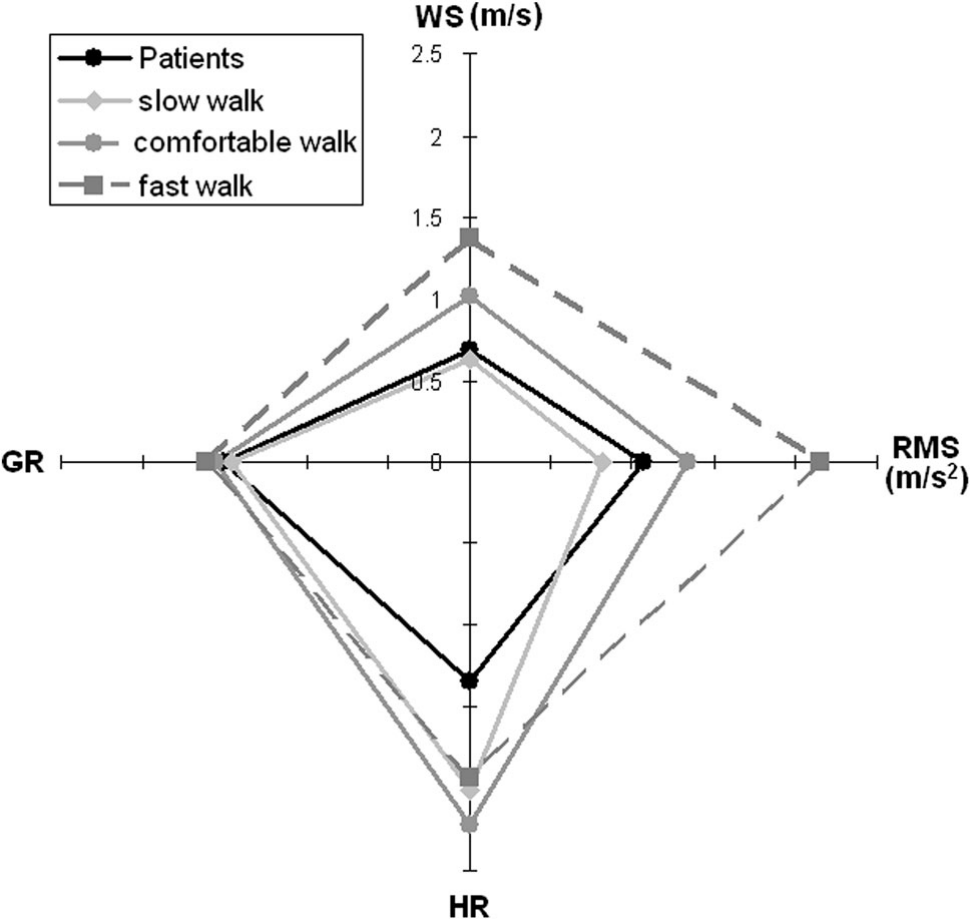
Mean values of walking speed (WS), RMS acceleration, harmonic ratio (HR), and golden ratio (GR: cycles/stance) for patients (*black line*, *circle markers*) and healthy subjects walking at slow (*light grey lines*), comfortable (*solid grey lines*), and fast speeds (*dotted grey lines*)

Figure 5 summarizes the results of the mean RMS acceleration and mean HR along the three body axes together with the mean speed and mean GR_0_.

## Discussion

The aim of this study was to analyze the stability and harmony of the gait of patients with subacute stroke with respect to those of healthy subjects at matched WS. Many differences were found when both groups walked at their comfortable speed, in accordance with the literature. Less obvious but more meaningful differences were observed when patients’ data were compared to those of slow-walking healthy subjects. The results are summarized in Fig. 5. The speed of patients was similar to the slow speed selected by healthy subjects, allowing for an interesting speed-matched comparison. Despite this similarity, patients showed higher instabilities and lower HR values. This last parameter was maximized by healthy subjects at their comfortable speed. GR was related to speed, but patients did not show alterations higher than those observed in slow-walking healthy subjects.

Upright gait stability was mainly altered along the LL axis for patients, with significantly higher acceleration values than those observed in slow-walking healthy subjects. Previous studies reported that normalized accelerations were significantly higher in patients with stroke compared to those of healthy controls along all three body axes [14]. A significant difference was also found in another study in which the AP RMS acceleration was expressed in terms of the percentage of CC RMS acceleration [13], and is hence independent of the type of normalization adopted. The present study found significant differences along AP and CC only when patients were compared with healthy subjects walking at their comfortable speed, whereas the speed-matched comparison highlighted a significant difference only along the LL axis. This discrepancy may be due to the fact that patients enrolled in the two studies cited above [13, 14] were more severely affected than those enrolled in the present one and/or to the normalization procedure applied in those studies. However, differences along AP and CC were also found in our study when patients’ data were compared with those of healthy subjects walking at a comfortable speed.

Menz et al. [11] reported that for healthy subjects, comfortable speed was the condition that maximized HR, especially along AP and CC. We also found a reduction of HR along AP and CC axes both at slower and faster speeds compared to the comfortable speed (as shown in Fig. 2); however, the high variability in the data prevented the effect of speed on HR from becoming statistically significant (Table 2). This absence of statistical significance may be due to differences in age and speed of our sample with respect to those in Menz et al.’ study (we enrolled a sample of elderly subjects, whose comfortable speed was 1.01 ± 0.17 m/s and fast one was 1.37 ± 1.17 m/s, whereas the subjects enrolled in their study were between 22 and 39 years old and had a comfortable walking speed of 1.31 ± 0.22 m/s).

A previous study showed a reduction in terms of HR in patients with respect to healthy subjects walking at their comfortable speed along CC and AP [13]. Our results confirm this trend. We performed a further analysis of harmonic relative powers, which revealed an association between LL and AP axes in patients, that could be interpreted as a possible coupling. In healthy subjects, the power of harmonics recorded in the sagittal plane (obtained by coupling AP and CC axes) was uncoupled from the LL one. In contrast, the harmonics observed along AP and LL were significantly correlated in patients. The reduction of power of higher-order harmonics is in line with the idea that disease states are often marked by less complex dynamics than those observed under physiological conditions, usually self-organized in complex harmonic structures [28].

One of these structures is the intrinsic harmony underlying the self-proportionality of the different phases that form a gait cycle. The self-similarity of GRs was lost in patients, but also in the elderly when walking at slow speed and even at comfortable speed when lower than 1 m/s. It should be noted that this limit is lower than the common WS of healthy adults. This is conceivably due to an age effect [6]. In fact, the speed chosen for fast walking by our healthy elderly was close to the conventional comfortable speed of young adults observed in the study of Menz et al. [11]. Our results showed that the three GRs converged towards the theoretical value of ϕ for WS > 1 m/s. This result is different from that found in patients with Parkinson’s disease, in which the alteration of GR was present even in patients with a walking speed of higher than 1 m/s [29]. Intriguingly, the GR values recorded for our patients with stroke were between those recorded at slow and comfortable speeds for healthy subjects. It can be concluded that the loss of the harmonic structure of walking is mainly attributable to slow speed, both for healthy elderly and patients. This suggests a possible role of a pendular dynamic mechanism of gait that exploits inertia and gravity for generating a movement of body segments embedded into a specific harmonic structure. This is in accordance with a recent study that showed that the connection between GR and anthropometric proportion (both related to the golden ratio) depends on the pendular mechanism of walking [30]. In healthy subjects, muscle activations intervene at discrete times to drive the pendular oscillations of the system for compensating for the small loss of energy. Different from patients with Parkinson’s disease, in which the alteration of GR seems related to the deficits in producing timed internal cues [29], it is conceivable that in patients with stroke, the main problem is not related to the timing of locomotor control, but to the force deficit that does not allow them to exploit the pendular mechanism of walking for generating harmonic movements. Hence, for patients with stroke, the GR alteration seems to be an effect, and not a cause, of speed reduction.

It should be noted that the patients with stroke enrolled in our study were mildly affected, being able to walk independently. Other neurological diseases, with different etiopathology such as Parkinson’s disease or ataxia expose subjects to wider alterations in terms of gait harmony [29].

From a neurorehabilitative point of view, our study focused attention on the upper part of the body of patients with stroke. Although harmony was not found to be greatly reduced in terms of proportions between gait phases, it was found to be altered in terms of trunk movements, with a reduced symmetric alternation between odd and even harmonics and with a pathological coupling between movements performed in the AP and LL directions. This is likely due to compensation strategies that on one hand may allow an increase gait ability but on the other hand may reduce gait stability, especially along the LL axis.

One of the aims of physical therapy is a good recovery of balance in subjects with stroke. Our study found that the main instabilities occur along the LL axis, and that these movements are coupled to those occurring along the AP axis. Hence, rehabilitation should decouple these movements, reducing the compensation strategies that lead to higher instabilities and hence a higher exposure to the risk of falling. Rehabilitation should also aim at favoring the reacquisition of adequate joint ranges of motion and muscle function on the paretic side, which may allow patients to use the correct alternation of contralateral movements, in turn allowing them to use the pendular mechanism of walking.

The main limits of this study are a small sample size and the fact that, for safety reasons, we did not ask to patients to walk at speeds other than their comfortable speed. However, the sample size was sufficiently high to identify many statistically significant differences. Another limit that may limit the generalizability of our results was the fact that all the enrolled patients were in the subacute phase of stroke; different results could be obtained for subjects in the chronic stage.

## Conclusion

This study analyzed gait features in patients with subacute stroke with respect to the values observed in age- and speed-matched healthy subjects. The results show that patients with stroke had LL instabilities due to a coupling of movements in the AP and LL axes. This could be due to compensation strategies. Rehabilitation should manage the trade-off between the risk of reduced stability and hence of falls and the return in terms of mobility. Furthermore, clinical staff can benefit from a low-cost quantitative assessment of gait benchmarks. The reduced walking speed of patients should be taken into account when comparing their data with those of slow-walking healthy adults.
